# Practice of over-the-counter dispensary of antibiotics for childhood illnesses in Addis Ababa, Ethiopia: a simulated patient encounter study

**DOI:** 10.1186/s13756-019-0571-x

**Published:** 2019-07-16

**Authors:** Eyosait Mekonnen Koji, Gebremedhin Beedemariam Gebretekle, Tinsae Alemayehu Tekle

**Affiliations:** 1 Department of Pediatrics and Child Health, College of Health Sciences, Addis Ababa University, Addis Ababa, Ethiopia; 2 School of Pharmacy College of Health Sciences, Addis Ababa University, Addis Ababa, Ethiopia

**Keywords:** Over-the-counter, Antibiotics, Non-prescription, Dispensary, Pharmacy, Medicine retail outlet, Ethiopia

## Abstract

**Background:**

Dispensary and use of antibiotics without prescriptions from qualified providers is a common practice in countries with poor pharmaceutical regulations and where due focus is not given to rational use. This practice is a main factor for the spread of antimicrobial resistance due to its non-reliance on pre-treatment microbiologic work-up, improper indications and dosing errors. This study was conducted to determine the rate of over-the-counter dispensary of antibiotics for common childhood illnesses among privately owned medicine retail outlets in Addis Ababa, Ethiopia.

**Methods:**

Pre-determined simulated patient visits depicting common childhood illnesses were employed to request antibiotics without prescriptions. A simple random sampling was used to select medicine retail outlets within Addis Ababa city. Trained data collectors filled structured data forms (including antibiotic requested, reasons for denial of dispense and details enquired by pharmacist) shortly after each patient enactment. Multi-variable logistic regression analysis was employed to explore factors associated with over-the-counter sales of antibiotics.

**Results:**

A total of 262 simulated encounters were surveyed. Of the 262 verbal antibiotic requests, 63.4% were dispensed. Close to 60% of encounters were accompanied by questions about a doctor’s visit or the child’s symptomatology while a past history of drug allergies was enquired in only 11.1% of visits. Over-the-counter dispensary was more likely when dispenser queried about symptoms was made (AOR: 2.412, 95%CI: 1.236, 4.706), for requests for more than one antibiotics (AOR: 2.988, 95%CI: 1.258, 7.095) and for simulated patient demands for oral antibiotics for children with acute diarrhea (AOR: 3.297, 95%CI: 1.248, 8.712) and parenteral antibiotics for those reported to receive in-patient care for pneumonia (4.516, 95% CI: 1.720, 11.857).

**Conclusions:**

The prevalence of providing antibiotics over-the-counter for pediatric illnesses in Addis Ababa is markedly high. Further studies into factors encouraging this malpractice are required. Enhancing education of personnel dispensing antibiotics and strict enforcement of national regulations are needed.

**Electronic supplementary material:**

The online version of this article (10.1186/s13756-019-0571-x) contains supplementary material, which is available to authorized users.

## Background

Since their discovery, antibiotics have revolutionized treatment of bacterial infections and have played a crucial role in reducing child mortality. Despite the enormous advances, infectious illnesses account 25% of deaths worldwide and 45% of mortality in low-income countries [1].High rates of resistance to antibiotics in sub-Saharan countries are leading to increased morbidity and mortality and enforcing higher costs on already strained health systems [2]. It is therefore high time to devise mechanisms to ensure the rational use of the available antibiotics.

A rational use of antibiotics relies on appropriate indications dependent on microbiologic yield, using for recommended durations of treatment, compliant patients, sound infection prevention practices and a well-regulated pharmaceutical sector. But both judicious and irresponsible consumption of antibiotics have markedly increased over the past decades. Antibiotics now represent the single largest group of drugs purchased in developing countries [3].

Self-medication with antibiotics is a widely witnessed phenomenon driving unregulated consumption and community antimicrobial resistance in low and middle income countries. Antibiotics are made available for members of communities making specific verbal requests for commonly recognized drugs [4–6]. Such non-prescription sale of antibiotics is associated with incomplete or shorter treatment courses and inappropriate drug and dose choices. This leads to selective pressure resulting in antibiotic-resistant strains adding to the increased financial burden posed on health systems. Children in particular are prone to acute and chronic adverse effects of such a malpractice [7]. Though a common observation, numerical data on the extent of the problem concerning common pediatric illnesses in Ethiopia is lacking. Hence, the aim of our study was to explore the prevalence of over the-counter (OTC) sales of antibiotics for childhood illnesses without prescriptions to be used as evidence in informing public education and upholding dispensary regulations.

## Methods

### Study setting and period

The study was conducted in Addis Ababa, capital city of Ethiopia. Administratively, it has ten sub-cities. Addis Ababa has 33 governmental and non-governmental hospitals, 94 health centers, 777 privately owned medical centers and 514 private medicine retail outlets (MROs). The study was undertaken from January to May 2018.

### Study design

A prospective, cross-sectional non-interventional study was done based on pre-determined simulated patient visits depicting common childhood illnesses to request antibiotics without prescriptions. Privately owned MROs were included for the study population while government affiliated facilities were excluded. The governmental outlets were excluded because of being few (*n* = 13) and since they were known not to dispense antibiotics without prescriptions.

### Sample size

An initial sample size was calculated using single proportion formula [7] taking a prevalence reference value (p) of 0.559, z of 1.96 and w of 0.05. A re-estimation was made as the source population had less than 10,000 units (n’ being the sample size extracted from the first formula and N being the source population). A final sample of 262 pharmacies or drug stores was calculated. Simple random sampling was used to enroll the required number of pharmacies and drug stores to the study.

### Data collection methods

Five sets of simulated patient encounters were formulated (Additional file 1). The simulated client visit was done using trained final year medical students, who enacted the encounters posing as a caretaker and responding to questions raised by the dispenser (if any). The pre- designed fictitious encounters I and II were based on observing a customary practice by parents or caretakers of visiting pharmacies and requesting self-medications for their children.

Scenarios III to V were prepared based on routine observations of parents of children receiving in-patient care at governmental hospitals being requested to visit privately owned outlets to buy medications unavailable within the governmental centers (Details provided in Additional file 1).

Simulation I: for a child with symptoms of a common cold and the caretaker requesting for Amoxicillin, Amoxicillin-clavulanate or Azithromycin.

Simulation II: for a child with symptoms of acute onset diarrhea and the caretaker requesting for Trimethoprim-Sulfamethoxazole or Metronidazole.

Simulation III: for an admitted child with pneumonia and parent requesting for Ceftriaxone, Cloxacillin or Vancomycin.

Simulation IV: for an admitted infant with meningitis and the parent requesting for Ampicillin, Cefotaxime or Gentamicin.

Simulation V: for a critically sick admitted child and the parent requesting for Ceftazidime, Cefepime or Meropenem.

There was single visit to a pharmacy or drug store in order to maintain data accuracy and avoid suspicion of dispensing personnel. The frequency of requests for the five clinical scenarios was evenly distributed. Testing of each clinical scenario was performed on single and multiple antibiotic requests for each of the categories I to V.

Shortly after each visit, a structured data collection form was filled concerning the encounter out of sight from the pharmacy’s or drug store’s staff. For each encounter, data consisting of the enacted visit, whether dispensers agreed to provide requested antibiotics without an available prescription, if further queries were made by dispenser - clinical presentation of the child, history of drug allergy, confirmation of a doctor’s visit, type of antibiotic offered for dispense, etc).

We defined an encounter which resulted in OTC dispense as one involving the dispenser agreeing to supply antibiotics without prescription (with or without stating the price), or willing to provide at a later date because of current unavailability after a verbal request from the simulator client. Daily supervisions and team discussions were used to maintain quality of data.

### Statistical analysis

The data were entered and analyzed using SPSS 25.0 statistical software. Descriptive statistics (frequency and percentage) were used to describe the events. Multi-variable logistic regression analysis was used to explore the association of OTC dispensary and independent variables. Statistical significance was set at *P*-value of 0.05.

### Ethical considerations

The study protocol was reviewed and approved by the Research and Publications Committee of the Department of Pediatrics and Child health, College of health sciences, Addis Ababa University.

## Results

A total of 262 pharmacies and drug stores were included in this study. An analysis of the responses for the simulated encounters showed that 166 (63.4%) of encounters ended up by an over-the-counter dispensary of requested antibiotics. Very few dispensers enquired about past history of drug allergy (11.1%) while 62.6% asked whether a doctor’s visit had taken place (Table 1).

**Table 1:** Baseline characteristics of simulated patient encounters in medicine retail outlets in Addis Ababa, Ethiopia

Variable	Frequency	Percentage (%)
Clinical scenario		
Scenario I	50	19.1
Scenario II	57	21.8
Scenario III	55	21.0
Scenario IV	50	19.1
Scenario V	50	19.1
Did dispenser (pharmacist or druggist) agree to dispense requested antibiotics?		
Yes	166	63.4
No	96	36.6
Did dispenser ask if a doctor’s visit had taken place?		
Yes	164	62.6
No	98	37.4
Was a history of drug allergy queried by dispenser?		
Yes	29	11.1
No	233	88.9
Did dispenser ask about the child’s symptoms?		
Yes	107	40.8
No	155	59.2

A detailed look of the 166 agreed-upon supplies for antibiotics without prescriptions showed that in 111 encounters (42.4%), dispensers physically produced the requested antibiotics with no prerequisites named; 16 (6.1%) demanded payment to be made first but agreed to dispense; and in 31 (11.8%) encounters, dispensers replied “We have run out of the requested antibiotics but we will make them available on another date”. In eight encounters (3.1%), the details of the encounter were undocumented. Only 96 simulated encounters with antibiotic requests (36.6% overall) were denied on the grounds of a lack of a prescription.

### Factors associated with over-the-counter dispensary of antibiotics

Request for an over-the-counter dispense of an antibiotic was significantly more likely to be approved when visits were accompanied by dispensers asking about the child’s symptoms (AOR: 2.412, 95%CI: 1.236, 4.706). The likelihood of over-the-counter dispensing was also three times more likely for requests of more than one antibiotic (AOR: 2.988, 95%CI: 1.258, 7.095). Rates of over the counter dispense for antibiotics taken per os and parenteral were comparable (64.5% versus 62.6% respectively) (Table 2) (Additional file 2).

**Table 2:** Factors associated with over-the-counter sales of antibiotics for simulated childhood illnesses in community pharmacies of Addis Ababa, Ethiopia

Variable		OTC dispense		Odds ratio (95% CI)	
		Yes	No	Crude odds ratio (COR)	Adjusted odds ratio (AOR)
Category of simulated encounter	I	31	19	1.00	1.00
	II	39	18	1.328 (0.597, 2.952)	3.297 (1.248, 8.712)*
	III	43	12	2.196 (0.932, 5.178)	4.516 (1.720, 11.857)*
	IV	23	27	0.522 (0.235, 1.159)	.504 (.219, 1.161)
	V	30	20	0.919 (0.411, 2.054)	.944 (.409, 2.175)
No. of antibiotics requested per encounter	Single	148	80	1.644 (0.795, 3.400)	2.988 (1.258, 7.095)*
	2 or more	18	16	1.00	1.00
Was query made of a Doctor’s visit?	Yes	104	60	1.006 (0.599, 1.692)	1.109 (.612, 2.011)
	No	62	36	1.00	1.00
Was a history of drug allergy asked?	Yes	19	10	1.112 (0.492, 2.50)	.553 (.226, 1.352)
	No	147	86	1.00	1.00
Did dispenser ask about child’s symptoms?	Yes	70	37	1.163 (0.696, 1.943)	2.412 (1.236, 4.706)*
	No	96	59	1.00	1.00

* Significant at *p* < 0.05

The likelihood for agreement to dispense antibiotics without prescription was significantly higher for simulations of out-patient requests to treat children with acute diarrhea (Scenario II: Oral Metronidazole and Trimethoprim-Sulfamethoxazole) (AOR: 3.297, 95%CI: 1.248, 8.712) and for in-patient care for pneumonia (Scenario III: Parenteral Ceftriaxone, Cloxacillin and Vancomycin) (4.516, 95% CI: 1.720, 11.857) (Table 2).

## Discussion

The global pooled prevalence of people accessing antibiotics following verbal requests was recently determined to be 78% with the highest rates being in South America. The practice is more often seen with symptoms of urinary tract and upper respiratory tract infections and for Penicillins and Fluoroquinolones [2]. Although non-prescription sales of antibiotics in Ethiopia is illegal, we found that the proportion of agreed upon encounters for over-the-counter supply of antibiotics for sick children was 63.4%. In a study conducted in Gondar, Ethiopia, 80% of simulated visits by mothers asking antibiotics for their sick children with acute diarrhea ended up with OTC antibiotic supply [7]. A similar multi-center Chinese study on childhood diarrhea was noted to have less rates of concurrence for an OTC sale of antibiotics for a sick child (55.9%) than ours [8]. Table 3 compares our findings with those of similar simulated studies conducted on pediatric illnesses in other countries.

**Table 3:** A comparison of prevalence of OTC dispensary of antibiotics based on simulated patient visits

Authors	Country	Simulated pediatric illness scenarios	Findings	Ref.
Diwan et al	India	Pediatric diarrhea	40.2%	[9]
Saengcharoen & Lerkiatbundit	Thailand	“	52.2%	[10]
Chang et al	China	“	55.9%	[8]
Shet et al	India	“	63.0%	[11]
^a^Mekonnen, Gebretekle & Alemayehu	Ethiopia	Multiple scenarios	63.4%	
Erku et al	Ethiopia	“	80%	[7]
Chuc et al	Vietnam	Uncomplicated upper respiratory tract infection	94.8%	[12]

^a^Our study

In parallel with the lack of adherence to “dispense with prescriptions”, symptomatology of the sick child was asked by 40.8% of personnel in the medicine retail outlets and the presence of a drug allergy history queried by 11.1%. This compares poorly with the aforementioned Chinese study with 64% of dispensers demanding to know about the clinical history of the children and 85% enquiring about a history of drug allergy [8]. Insistence on stating that prescriptions made after doctors’ visits being mandatory for dispensary was perceived to be low. A cross-sectional study based on simulated visits conducted in Sri Lanka showed that recommendations to visit doctors were made in only 7% of instances [13].

We observed that requests for more than one antibiotic were more likely to result in an OTC dispensary of antibiotics. The same was true when dispensers enquired about child’s symptoms. Chang et al’s study on community pharmacies in three Chinese urban centers showed that geographical location of pharmacies (among the three study areas) and the presence of a licensed pharmacist were determinants of dispensing antibiotics without prescriptions. Similarly, in a Spanish study performed by adult patient simulations of respiratory infections, a rural or semi-rural setting for pharmacies was more likely for obtaining antibiotics without prescriptions. Alele et al’s study from Eastern Uganda showed that higher socioeconomic status and educational level of parents and children aged 3–5 years correlated significantly with OTC supply of antibiotics for sick children. Complaints of cough and fever accounted for most approved self-antibiotics for children less than 5 years [5, 8, 14].

Further, we noted that requests for OTC for oral antibiotics for simulations of children with acute diarrhea (Trimethoprim-Sulfamethoxazole and Metronidazole) and for parenteral antibiotics for simulated cases of children admitted for pneumonia (Ceftriaxone, Cloxacillin or Vancomycin) were more likely to be approved by dispensers. Pharmacists in Sri Lanka were more likely to dispense oral Ciprofloxacin (70% of requests approved) when faced with requests without prescriptions, similar to those in the states of Madhya Pradesh and Karnataka, India [9, 11, 13].

In North-eastern Tanzania, Combination preparations of Ampicillin and Cloxacillin (AmpiClox) and Azithromycin were most commonly associated with OTC dispensary for adults with respiratory and urinary complaints respectively [15].

The design of these fictitious encounters was based on two routine observations made by investigators. The first concerns the preference of parents or caretakers to visit medicine retail outlets and request OTC antibiotics for their sick children rather than visiting a physician. The second is the practice of caretakers being given prescriptions for antibiotics (which government hospital pharmacies have run out of) to be purchased from privately owned outlets. Hence, they confirm to realistic observations within Addis Ababa. This was reaffirmed by a rarely witnessed wariness on the part of dispensers. These observed practices are also common around Ethiopia. Hence, one may argue that our conclusions are reflective of the nationwide problem. Indeed, findings may be under-estimated as the further away from administrative centers and urban areas, the less stringent enforcement and adherence to regulations are. A limitation to our study design is that we did not take into consideration whether dispenser was a pharmacist, druggist or indeed in the worst scenarios, an untrained professional. This may influence the OTC dispense for self-medication requests. Another factor which may change outcomes is the proximity of medicine retail outlets to hospitals.

## Conclusions

In summary, the prevalence of over the counter dispense for antibiotics for childhood illnesses is very high in Addis Ababa. The high rate of OTC dispensary was significantly related to requests made by dispensers regarding symptomatology, the number of requested antibiotics and simulated clinical presentations. Additional researches are needed on identifying further underlying factors. Existing policies need to target enhanced enforcement of only standard prescription-based dispensaries. Public education and training programs for pharmacy staff to promote the appropriate use of antibiotics are warranted.

## Additional file

Additional file 1: Simulated patient encounters. (DOCX 19 kb)

Additional file 2: Raw data. (SAV 4 kb)

## Data Availability

Available.

## Abbreviations

AOR: Adjusted odds ratio
COR: Crude odds ratio
MROs: Medicine retail outlets (MROs)
OTC: Over-the-counter
