# Supplementary material for: Practice of over-the-counter dispensary of antibiotics for childhood illnesses in Addis Ababa, Ethiopia: a simulated patient encounter study
Source: Antimicrob Resist Infect Control. 2019 Jul 16;8:119. doi: 10.1186/s13756-019-0571-x (PMC6636089; doi:10.1186/s13756-019-0571-x)
Supplement: Supplementary file 1 — Simulated patient encounters. (DOCX 19 kb) [file 13756_2019_571_MOESM1_ESM.docx]

# **Additional file 1**

# **Clinical scenarios**

# **Clinical scenario A: Upper respiratory tract infection**

The SC will pose as the caretaker of a one year old child seeking care for the child who has

symptoms of cough, fever and a runny nose. Antibiotics included for assessment by this clinical

scenario were Amoxicillin, Amoxicillin-clavulanate and Azithromycin.

The SC first says to dispenser ‘My one year old son has had a runny nose, cough and a low

grade fever. Could you give me medicine X (one of the three listed above) to cure his

symptoms?’

*Answers to questions likely to be asked by the dispenser were predefined as follows:*

In response to questions about symptoms (if any), the SC answers ‘The child has been having a

runny nose, occasional cough and a low grade fever of one day. There is no grunting or

interruptions of feeding. He is playful and sleeps well’.

In response to questions about a history of drug allergy (if any), the SC answers ‘No’.

In response to a request for a prescription (if any), the SC says ‘I do not have a prescription’.

In response to questions about whether the caretaker has taken his/her son for a visit to a

doctor (if any), the SC answers ‘No, I have not’.

In response to an insistence to visit a doctor/clinic, the SC says ‘I think it’s just a minor illness. I

don’t think it requires a visit to a doctor. Could you just give me the medication X as my son had

a similar illness before and this medication X had made him feel much better (specify the name

of the drug from the three listed above)?

#

# **Clinical scenario B: Diarrhea**

The SC will pose as the caretaker of an eighteen month old child seeking care for an acute

onset diarrhea. Antibiotics included for assessment by this clinical scenario are

Trimethoprim-Sulfamethoxazole and Metronidazole.

The SC first says to the dispenser ‘My 18 month old daughter has had diarrhea and a low grade

fever. The child feeds well but has had a few episodes of vomiting. Could you give me medicine

X (one of the two listed above) to cure her symptoms?’

*Answers to questions likely to be asked by the dispenser were predefined as follows:*

In response to questions about symptoms (if any), the SC answers ‘The child has been having

diarrhea and a low grade fever. She feeds well but has had a few episodes of vomiting’.

In response to questions about a history of drug allergy (if any), the SC answers ‘No’.

In response to a request for a prescription (if any), the SC says ‘I do not have a prescription’.

In response to questions about whether the caretaker has taken his/her daughter for a visit to a

doctor (if any), the SC answers ‘No, I have not’.

In response to an insistence to visit a doctor/clinic, the SC says ‘I think it’s just a minor illness. I

don’t think it requires a visit to a doctor. Could you just give me the medication X as my

daughter had a similar illness before and this medication X had made her feel much better

(specify the name of the drug from the two listed above)?

# **Clinical scenario C: Pneumonia (In-patient)**

The SC will pose as the caretaker of a 2 year old boy seeking parenteral medications.

Antibiotics included for assessment by this clinical scenario are Ceftriaxone, Cloxacillin and

Vancomycin.

The SC first says to the dispenser ‘My 2 year old son has been having cough, fast breathing

and a high grade fever.’ Could you please give me the medication X (one of the three listed

above)?

*Answers to questions likely to be asked by the dispenser were predefined as follows:*

In response to questions about symptoms (if any), the SC answers ‘He has been having cough,

fast breathing, a high grade fever, grunting and poor appetite’.

In response to questions about a history of drug allergy (if any), the SC answers ‘No’.

In response to a request for a prescription (if any), the SC says ‘I have lost my prescription but

the treating physician has asked me for ‘X’ number of vials of the specified medication’.

In response to questions about whether the caretaker has taken his/her son for a visit to a

doctor (if any), the SC answers ‘Yes, I have. He has been admitted to a hospital and has

been receiving intranasal oxygen and injectable medications. He is not feeding well orally. He

had been receiving medication X but the hospital has run out of it. Can you give me ‘x’ number

of vials of the specified medication to ensure continuity of his treatment?’.

# **Clinical scenario D: Meningitis (In-patient)**

The SC will pose as the caretaker of a 1 month old female infant seeking parenteral

medications. Antibiotics included for assessment by this clinical scenario are Gentamicin,

Cefotaxime and Ampicillin.

The SC first says to the dispenser ‘My 1 month old girl has been having poor feeding and a high

grade fever.’ Could you please give me the medication X (one of the three listed above)?

*Answers to questions likely to be asked by the dispenser were predefined as follows:*

In response to questions about symptoms (if any), the SC answers ‘She has been having poor

feeding, a high grade fever, occasional vomiting of ingested milk and fast breathing’.

In response to questions about a history of drug allergy (if any), the SC answers ‘No’.

In response to a request for a prescription (if any), the SC says ‘I have lost my prescription but

the treating physician has asked me for ‘X’ number of vials of the specified medication’.

In response to questions about whether the caretaker has taken his/her daughter for a visit to a

doctor (if any), the SC answers ‘Yes, I have. She has been admitted to a hospital and has

been receiving injectable medications. She is feeding poorly. She had been receiving

medication X but the hospital has run out of it. Can you give me ‘x’ number of vials of the

specified medication to ensure continuity of her treatment?’.

# **Clinical scenario E: Sepsis (In-patient)**

The SC will pose as the caretaker of a 2 year old boy seeking parenteral medications.

Antibiotics included for assessment by this clinical scenario are Meropenem, Ceftazidime and

Cefepime.

The SC first says to the dispenser ‘My 2 year old boy has been having a high grade fever and

has lost consciousness.’ Could you please give me the medication X (one of the three listed

above)?

*Answers to questions likely to be asked by the dispenser were predefined as follows:*

In response to questions about symptoms (if any), the SC answers ‘He has been having poor

feeding and a high grade fever for two days but has lost consciousness since yesterday’.

In response to questions about a history of drug allergy (if any), the SC answers ‘No’.

In response to a request for a prescription (if any), the SC says ‘I have lost my prescription but

the treating physician has asked me for ‘X’ number of vials of the specified medication’.

In response to questions about whether the caretaker has taken his/her son for a visit to a

doctor (if any), the SC answers ‘Yes, I have. He has been admitted to a hospital and has been

receiving intranasal oxygen, intravenous fluids and injectable medications. He had been

receiving medication X but the hospital has run out of it. Can you give me ‘x’ number of vials of

the specified medication to ensure continuity of his treatment?’
